# Aberrant Disgust Responses and Immune Reactivity in Cocaine-Dependent Men

**DOI:** 10.1016/j.biopsych.2013.08.004

**Published:** 2014-01-15

**Authors:** Karen D. Ersche, Cindy C. Hagan, Dana G. Smith, Sanja Abbott, P. Simon Jones, Annemieke M. Apergis-Schoute, Rainer Döffinger

**Affiliations:** a Department of Psychiatry, University of Cambridge, Addenbrooke’s Hospital, Cambridge, United Kingdom; b Behavioural and Clinical Neuroscience Institute, University of Cambridge, Addenbrooke’s Hospital, Cambridge, United Kingdom; c Department of Clinical Biochemistry and Immunology, Cambridge University Hospitals National Health Service Foundation Trust, Addenbrooke’s Hospital, Cambridge, United Kingdom

**Keywords:** Conditioned immunoactivation, cytokines, drug addiction, interleukin (IL)-6, infection susceptibility, interoception

## Abstract

**Background:**

Infectious diseases are the most common and cost-intensive health complications associated with drug addiction. There is wide belief that drug-dependent individuals expose themselves more regularly to disease-related pathogens through risky behaviors such as sharing pipes and needles, thereby increasing their risk for contracting an infectious disease. However, evidence is emerging indicating that not only lifestyle but also the immunomodulatory effects of addictive drugs, such as cocaine, may account for their high infection risk. As feelings of disgust are thought to be an important psychological mechanism in avoiding the exposure to pathogens, we sought to investigate behavioral, physiological, and immune responses to disgust-evoking cues in both cocaine-dependent and healthy men.

**Methods:**

All participants (*N* = 61) were exposed to neutral and disgust-evoking photographs depicting food and nonfood images while response accuracy, latency, and skin conductivity were recorded. Saliva samples were collected before and after exposure to neutral and disgusting images, respectively. Attitudes toward disgust and hygiene behaviors were assessed using questionnaire measures.

**Results:**

Response times to disgust-evoking photographs were prolonged in all participants, and specifically in cocaine-dependent individuals. While viewing the disgusting images, cocaine-dependent individuals exhibited aberrant skin conductivity and increased the secretion of the salivary cytokine interleukin-6 relative to control participants.

**Conclusion:**

Our data provide evidence of a hypersensitivity to disgusting stimuli in cocaine-dependent individuals, possibly reflecting conditioned responses to noningestive sources of infection. Coupled with a lack of interoception of bodily signals, aberrant disgust responses might lead to increased infection susceptibility in affected individuals.

One of the most serious and costly health complications associated with drug addiction is the risk of contracting or transmitting infectious diseases (1–5). Reducing the disproportionately high rate of infection in chronic drug users has long been a priority target of harm-reduction policies (6). Although the introduction of needle-exchange schemes has been successful for intravenous cocaine users, harm reduction remains a challenge for users who snort cocaine or inhale crack-cocaine (7–9). For instance, hepatitis C rates of up to 80% have been estimated in crack-cocaine smokers (6). Risky behaviors such as sharing unsterile straws or pipes, engaging in unprotected sex, or personal hygiene inadequacies have been considered to account for the increased prevalence of infections in noninjecting users (10–12). Yet several studies have failed to find relationships between these risky behaviors and infection rates in this population (10,13), suggesting that factors other than lifestyle may account for their high infection risk.

Emerging evidence indicates that addictive drugs have immunomodulatory effects that may decrease drug users’ ability to fight infections (14–17). Cocaine has been shown to alter immune cell activity and cytokine production (18,19), leading to the suppression of innate immune responses (20,21). Conversely, cocaine has also been shown to prolong inflammation, possibly through neuroendocrine interactions (22), which could facilitate the development of systemic low-grade inflammation (23,24). Current strategies to tackle the high infection rates in chronic drug users focus primarily on remediating harm, although proactive approaches strengthening protective mechanisms might be more desirable.

Self-care behaviors are one vital means through which infectious diseases can be prevented. A critical mechanism underlying the development of avoidant and protective self-care strategies are learned relationships between cues signaling sickness, feelings of disgust, and unconditioned immune responses (25–27). Stimuli evoking feelings of disgust can induce bodily sensations of revulsion and nausea, eliciting a desire to withdraw from disgust-evoking cues (28,29). Stimuli that typically convey the so-called “core” or “pathogen disgust” are rotten food, decomposing organic matter, poor hygiene, and body products (30,31). Repeated encounters with disgusting stimuli, and the feelings of sickness they elicit, may be necessary to associate these evocative memory traces with avoidance or hygienic behaviors (27,32). As the central nervous system actively communicates with the immune system, Pavlovian conditioning to disgust-evoking cues can be observed in both behavioral and immune responses (26,33,34). For example, Schaller and colleagues examined blood samples of healthy volunteers who were exposed to either infection- or gun-related photographs (35). Subsequent in vitro stimulation of these blood samples with bacterial lipopolysaccharide (LPS) showed that samples provided by volunteers who were exposed to the disease-related photographs had a significantly greater increase in the cytokine interleukin-6 (IL-6) compared with those in the control condition. The pro-inflammatory cytokine IL-6 is a key regulator of inflammatory processes in response to acute infection (36;37). The authors suggested that participants’ prior experience with pathogens associated with the infectious diseases shown in the photographs may have triggered an anticipatory response that facilitated the release of IL-6 (35).

Several studies have shown that cocaine directly suppresses production of IL-6 in the blood during acute infection (20,21), but little is known of how cocaine-dependent individuals (CDIs) respond to cues signaling infection. Salivary immune responses are of particular interest because the mouth is a major gateway of microbial infection and mucosal immunity, which is independent from the rest of the peripheral lymphoid immunity (38). In the current study, we measured disgust-induced behavioral, physiological, and immune responses in CDIs and healthy volunteers to investigate two contrasting hypotheses of infection risk in CDIs. It is possible that CDIs are insensitive to disgust-evoking cues, thereby failing to anticipate risks of infection and exposing themselves to pathogens. If this hypothesis were correct, we would predict blunted disgust processing in the cocaine group, along with self-reports showing little reflection on disgusting experiences and poor hygienic practices. Alternatively, CDIs could show conditioned hypersensitive responses to disgust-evoking stimuli because of their frequent history of infection, but they fail to use this information appropriately to guide their behavior. If this hypothesis were correct, we would predict increased responses to stimuli predictive of infection in the cocaine group but no difference from the control group in terms of cognitive and behavioral strategies relating to risks of infection.

## Methods and Materials

### Participants

Sixty-five men were recruited within the local community upon referral from probation officers, health care professionals, advertisements, or word-of-mouth. For inclusion, participants had to be male, 20 to 60 years of age, and able to read and write in English. Drug-dependent volunteers had to satisfy the DSM-IV-TR (39) criteria for cocaine dependence, whereas healthy control volunteers were required to have no personal or family history of substance misuse disorders. Exclusion criteria for all volunteers included a lifetime history of a psychotic disorder, neurological illness or traumatic head injury, an autoimmune or metabolic disorder, or HIV infection. All volunteers consented in writing and were screened for current psychiatric disorders using the Mini-International Neuropsychiatric Inventory (40). Psychopathology in drug users was further evaluated using the Structured Clinical Interview for DSM-IV (41). Current negative emotional states were measured using the Depression Anxiety Stress Scale (DASS-21) (42); verbal IQ was estimated using the National Adult Reading Test (NART) (43). The protocol was approved by the National Research Ethics Committee (NREC10/H0306/69, Principal Investigator: K.D. Ersche) (44).

The 35 CDIs met the DSM-IV-TR criteria for cocaine dependence, but none were actively seeking treatment for cocaine use. Urine samples tested positive for stimulants in all but four users, indicating they had consumed cocaine or amphetamines within the past 72 hours (45). To avoid potentially confounding effects of drug abstinence, we restricted subsequent analysis to those with a stimulant-positive urine screen, leaving 31 CDIs in the sample. Participants reported using cocaine for an average of 15 years (±7.9 SD), mainly intranasally or by inhalation; approximately one quarter of users (23%) injected cocaine intravenously (Supplement 1). The majority of CDIs also met criteria for dependence on another substance (93% nicotine, 45% opiates, 29% alcohol, 23% cannabis, 3% amphetamines) and used other drugs sporadically (51% cannabis, 19% sedatives, 16% ecstasy, 6% opiates, 3% hallucinogens; see Supplement 1 for details on sporadic and prescription drug use).

Thirty healthy control participants were screened for drug and alcohol misuse, and none met criteria for abuse or dependence. Urine samples were negative for illicit substances in all individuals. Seventy percent were either past or current tobacco smokers, and 57% reported having social experiences with cannabis; none reported taking prescribed or illicit drugs on a regular basis.

### Procedures

Participants were assessed at the Wellcome Trust Clinical Research Facility, Addenbrooke’s Hospital, Cambridge, United Kingdom. Biological samples and fitness assessments were taken on arrival to establish health status. Urine samples were tested for current infection with cytomegalovirus (CMV) and Epstein-Barr virus, persistent viruses that affect the immune system (46). Blood samples were drawn to measure serum levels of C-reactive protein (CRP) as a marker of inflammation (47). We used saliva sampling as a noninvasive method to measure changes in cytokine levels before and after exposure to neutral and disgust-evoking photographs; a method that has been used successfully in previous studies (48,49). At three time points during their visit, participants were asked to rinse their mouth with water to provide a 2-mL sample of saliva by passive drool through a straw into a cryovial (http://www.salimetrics.com). Samples were provided on arrival (t_1_), immediately after exposure to neutral photographs (t_2_), and immediately after exposure to disgust-evoking photographs (t_3_); the samples were frozen at −80°C before being analyzed for the following cytokines: IL-6, IL-1beta (IL-1β), and tumor necrosis factor-alpha (TNF-α), which are typically induced together during an infectious challenge (36,37). We also examined interferon-gamma (IFN-γ) and IL-12, two TH1-cytokines with important roles in both cellular and innate immunity (50); the anti-inflammatory cytokine IL-10; and IL-8, a pro-inflammatory neutrophil chemotactic factor (51).

Before the exposure procedures, participants completed two questionnaires to assess interindividual differences in disgust reactions. The Disgust Propensity-Sensitivity Scale—Revised (52) is a trait measure of disgust, in which participants rated on a 5-point Likert scale the frequency of affective experiences of disgust and the cognitive evaluation of these experiences. The Hygiene Inventory (HI-23) (53) is a measure to assess various aspects of hygiene-related behaviors, including hand-washing, personal grooming, food handling, and household cleanliness.

### Evocative Task

Participants were shown 120 colored photographs, half depicting images of neutral valence (neutral foods, household items) and the other half showing disgusting images (rotten/moldy foods, dirty objects, dead bodies/animals, disease/injury, body products). The photographs were selected from a pool of 180 pictures either downloaded from the Internet or selected from the International Affective Picture Series (54). To ensure the correct valence classification, all pictures were rated for pleasantness, arousal, disgust, and nausea on a Likert scale (1 = not, 7 = very) by 15 healthy men before experimental testing (Table S1 in Supplement 1). The Wilcoxon signed-rank test confirmed that photographs in the neutral and disgusting categories differed significantly with regard to ratings of pleasantness, arousal, disgust, and nausea (all *p*s < .001).

**Figure 1:**
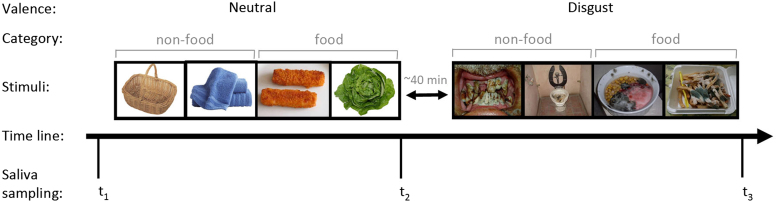
The evocative task includes 120 different photographs, divided into two segments according to their valance (neutral, disgust). The neutral segment always preceded the disgust segment by approximately 40 minutes. Each segment consisted of 30 photographs depicting different foods and 30 photographs depicting various nonfood items; 20 photographs from each category were shown twice, resulting in 100 trials per segment (50 food and 50 nonfood photographs). Participants were asked to indicate by button press whether they had seen the photograph before. Response time, detection accuracy and skin conductivity were recorded during each segment. For the collection of the cytokine data, saliva samples were collected over three time points: on arrival; immediately after the neutral segment; and immediately after the disgust segment.

The task was administered in two segments (neutral, disgust) with an intervening period of approximately 40 minutes (Figure 1). The neutral images always preceded the disgusting images to obviate a potential carryover effect of disgust-induced cytokine increases to the neutral condition. Each segment consisted of 100 trials (50 food, 50 nonfood) shown to participants in two separate blocks (food, nonfood). The differentiation between food and nonfood images reflected two routes of transmission: ingestion versus contact. Each block contained 30 images, during which a random selection of 20 images was shown twice. The order of the block to be shown first (food or nonfood) was randomized to control for order effects, and the presentation sequence of images within each block was pseudo-randomized to avoid consecutive repetition of the same image. Each photograph was displayed in the center of the computer screen for 6 seconds, followed by a 3-second interstimulus interval during which participants were asked to indicate by button press whether they had seen the picture before; a means to ensure that participants attended to the images. The 3-second interval was maintained irrespective of participants’ response speed. We recorded median latencies of correct responses and detection accuracy, which was calculated from the hit rate minus the false alarm rate divided by the number of trials (55). We sought to investigate differential responses to disgusting versus neutral images.

### Physiological Measures

Skin conductance responses (SCRs) were measured at 1000 Hz using a BiopacSystems operating AcqKnowledge-4.1 (MP36R, BiopacSystems, Santa Barbara, California). SCRs were identified within a 7-second window after the onset of each stimulus; minima were identified within the first 1.5 seconds and maxima between .5 and 7 seconds. To remove high-frequency spikes, data was zero-phase filtered within a time window of 1000 milliseconds. Low-pass filtering was applied within a time window of 200 milliseconds at a cutoff of .01, which provided a baseline for correcting phasic changes in SCRs. Reponses in microsiemens were locally normalized as a percentage of the mean of the phasic response within the 7-second window. Stimuli that elicited no positive change or a response of less than 2% were classed as generating no SCRs. Individuals’ median SCR of correct responses for each category and valence were calculated. Due to technical difficulties, complete data sets of both neutral and disgust segments were only available from 39 participants (20 controls, 19 cocaine) of 50 recorded data sets. Demographics of participants with missing data did not significantly differ from the rest of the sample.

### Statistical Data Analysis

Data were analyzed using Statistical Package for the Social Sciences v.20 (IBM SPSS Statistics, IBM Corporation, Armonk, New York). Independent sample *t* tests were used for group comparisons on demographic variables, vital signs (pulse, blood pressure), and CRP levels; for infection status, we used Fisher’s exact test. In preparation for parametric analyses, DASS-21 scores, CRP levels and cytokine data were square-root transformed to reduce skew (56); untransformed values are displayed in figures and tables. To obviate potential confounding effects of group differences in mood states and inflammatory markers on immune responses (57–59), DASS-21 total scores and CRP levels were included as covariates in all group comparisons. Intelligence levels (as indicated by NART scores) differed significantly between groups, yet the presence of a group interaction precluded the use of NART scores as a nuisance covariate (60).

We used multivariate analyses of covariance (ANCOVA) to examine group differences on the Disgust Propensity-Sensitivity Scale—Revised and Hygiene Inventory questionnaires. We subsequently applied separate repeated-measures ANCOVA models including accuracy and latency, or SCR data with stimulus category (food, nonfood) and stimulus valence (neutral, disgust), as the two within-subject factors and group (controls, cocaine) as the between-subject factor. The conservative Greenhouse-Geisser correction was used if the Mauchly’s test showed violations of sphericity.

For the cytokine analysis, we first compared salivary samples provided at t_1_ using multivariate ANCOVA to verify the absence of group differences at baseline. We then used repeated-measures ANCOVA to examine changes in cytokine levels before and after provocation procedures, with time-point (t_1_, t_2_, t_3_) as the within-subject factors and group as the between-subject factor. We implemented two separate contrasts in the model to directly compare the changes in cytokine levels between t_1_ versus t_2_ (neutral) and t_2_ versus t_3_ (disgust) where significant interactions were identified.

To obviate potentially confounding effects of concomitant medication, intravenous drug use, comorbid opiate dependence, and comorbid alcohol dependence, we reanalyzed the data excluding the following conditions: 1) volunteers who reported taking prescribed medication (*n* = 12), 2) individuals who were CMV-positive/invalid (*n* = 2), 3) intravenous drug users (*n* = 7), 4) individuals with comorbid opiate dependence (*n* = 14), or 5) individuals with comorbid alcohol dependence (*n* = 9). If not stated otherwise, Pearson’s correlation coefficient was calculated for descriptive purposes. All statistical tests were two-tailed, and significance levels were set at .05.

### Biomarker Analysis

Biological samples were analyzed at Addenbrooke’s Hospital Biomedical Campus (http://www.cuh.org.uk). Serum CRP levels were determined using the particle-enhanced turbidimetric immunoassay technique (RCRP-[DF34]Flex reagent cartridge; Dade-Behring, Milton-Keynes, United Kingdom). Salivary cytokines were measured using the MSD-7-plex-ultra-sensitive human pro-inflammatory cytokine kit (K15008C-2), which was supplied by MesoScale Discovery (Gaithersburg, Maryland). Urine samples were screened for the presence of CMV or Epstein-Barr virus using real-time Taqman polymerase chain reaction assays.

## Results

### Demographics and Clinical Variables

The groups were well matched for age (t_59_ = .46, *p* = .650), but CDIs had significantly lower NART scores compared with their healthy peers (t_43.8_ = .53, *p* < .001). The groups did not differ on vital signs, including pulse rate (t_59_ = 1.15, *p* = .253), systolic (t_59_ = –.19, *p* = .847) and diastolic blood pressure (t_59_ = .85, *p* = .399), indicating that the CDIs were not acutely intoxicated. CDIs, however, scored significantly higher on all three subscales of the DASS-21, reflecting increased levels of depressive mood (*F*_1,55_ = 35.8, *p* < .001), anxiety (*F*_1,55_ = 33.9, *p* < .001), and stress (*F*_1,55_ = 25.0, *p* < .001). The groups did not differ in terms of self-reported hygienic behaviors (*F*_1,53_ = 1.07, *p* = .305), disgust propensity (*F*_1,53_ = 1.17, *p* = .285) or disgust sensitivity (*F*_1,50_ = .36, *p* = .552).

Although a larger number of CDIs (19%) compared with control volunteers (7%) reported having more than three colds per year, this difference was not statistically significant (Fishers’ *p* = .255); information regarding the frequency of other infections was not collected. CDIs also reported having more difficulties fighting infections (Fishers’ *p* = .011) and used antibiotic medication more frequently (Fishers’ *p* = .053) compared with their healthy peers. For descriptive data, see Table S2 in Supplement 1. Laboratory tests revealed a positive CMV infection in one control volunteer and an invalid test in one drug user; no evidence was found of Epstein-Barr virus infection in either group. We measured increased CRP levels in blood serum in the cocaine group (mean 6.3 mg/L ± 6.1 SD) compared with the control group (mean 3.0 mg/L ± 3.8 SD; t_59_ = –3.13, *p* = .003).

### Disgust-induced Behavioral and Physiological Responses

**Figure 2:**
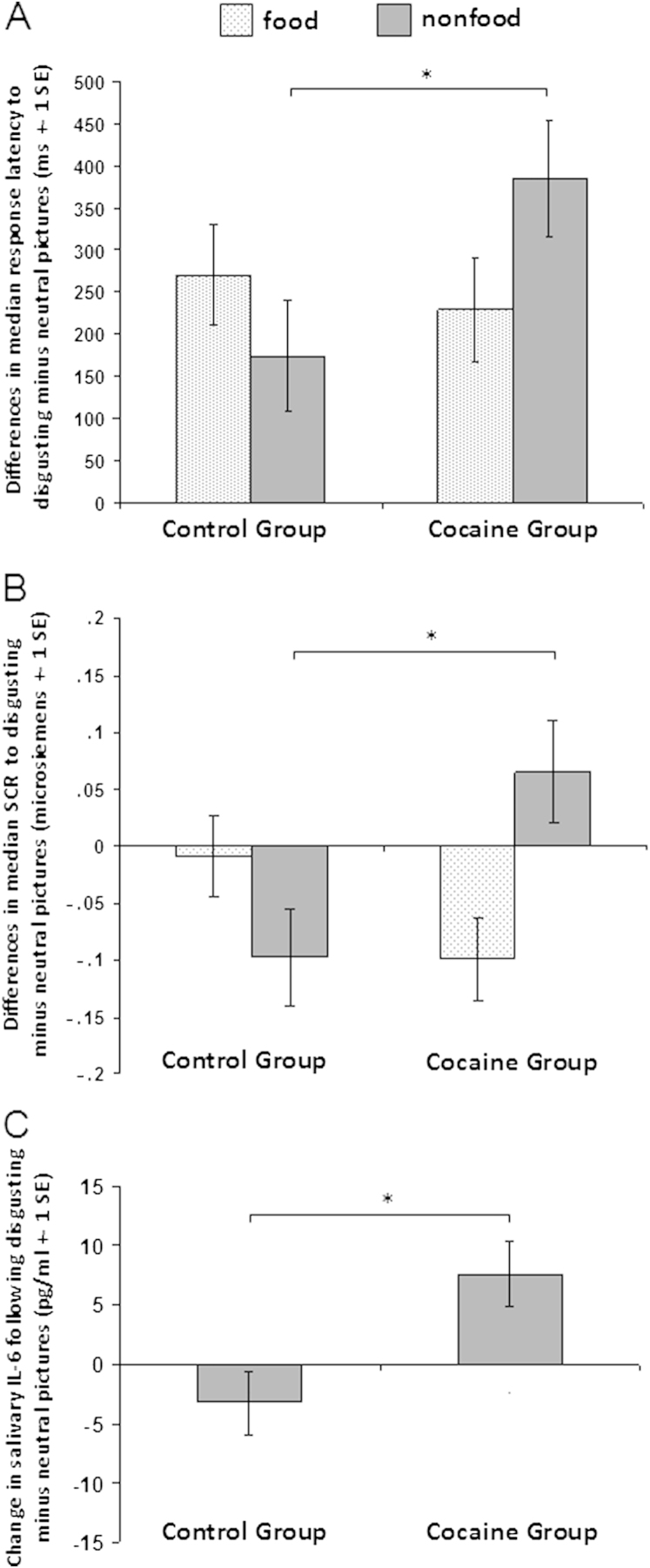
The graphs display differences between disgust minus neutral trials separately for food and nonfood pictures. **(A)** Disgust provocation prolonged the response times in all volunteers, and specifically in cocaine-dependent individuals, who showed a significant slowing in response speed during the presentation of disgusting nonfood pictures. **(B)** The viewing of disgusting nonfood pictures significantly increased SCRs in the cocaine group but not in control volunteers. **(C)** Cytokine levels were measured following the exposure to neutral and disgusting pictures but not following the food and nonfood blocks. In comparison to healthy volunteers, cocaine-dependent individuals showed a significant increase in interleukin-6 (IL-6) following provocation of disgust compared with the neutral condition. (The group comparison of salivary IL-6 does not include the data of the individual with overall extreme levels of pro-inflammatory cytokines).

Performance data and physiological responses are shown in Table S3 in Supplement 1. Response latencies were prolonged in all participants to disgusting images compared with neutral images, as reflected by a significant main effect of valence (*F*_1,48_ = 7.64, *p* = .008); no main effects of valance were observed for accuracy or SCRs. No main effects of group and stimulus category were found with regard to accuracy, latency, or SCRs. A significant three-way interaction of category-by-valence-by-group was identified on response latency (*F*_1,48_ = 4.59, *p* = .037; Figure 2A) due to CDIs exhibiting longer response times to disgusting nonfood pictures relative to neutral nonfood pictures compared with controls (*F*_1,50_ = 4.24, *p* = .045). Latency changes for food-related photographs were nonsignificant (*F*_1,49_ = .04, *p* = .838). There was also a significant three-way category-by-valence-by-group interaction on SCRs (*F*_1,35_ = 6.27, *p* = .017). CDIs showed significantly increased SCRs to disgusting nonfood relative to neutral nonfood images compared with control participants (*F*_1,35_ = 5.06, *p* = .031; Figure 2B); SCRs to food photographs were nonsignificant (*F*_1,35_ = 2.32, *p* = .137).

### Baseline and Disgust-induced Immune Responses

Descriptive data is shown in Table S4 in Supplement 1. Salivary cytokine samples provided on arrival did not differ between groups (*p* > .1), except for IL-6 (*F*_1,53_ = 6.14, *p* = .016), with baseline levels significantly increased in CDIs. Group comparisons across the three time-points revealed a significant main effect of time for IL-1β (*F*_2,106_ = 7.82, *p* = .001), TNF-α (*F*_1.5,78_ = 6.21, *p* = .007), IL-6 (*F*_1.3,68_ = 6.13, *p* = .010), IL-10 (*F*_1.6,84_ = 4.84, *p* = .016), and IFN-γ (*F*_1.7,90_ = 6.75, *p* = .003). A significant main effect of group emerged for IL-6 (*F*_1,53_ = 13.03, *p* = .001). We also identified a significant time-by-group interaction for IL-6 (*F*_1.3,68_ = 6.97, *p* = .006) and IL-1β (*F*_2,106_ = 4.42, *p* = .017). Post hoc contrasts confirmed that changes in IL-6 were significant between the neutral (t_2_) and disgust (t_3_) conditions (*F*_1,53_ = 6.06, *p* = .017) but not between arrival (t_1_) and the neutral (t_2_) condition (*F*_1,53_ = 1.16, *p* = .287). Post hoc contrasts for IL-1β were nonsignificant. On further inspection, one CDI showed increased overall levels in cytokine concentration for IL-10, IL-12p70, IL-6, and TNF-α, deviating by almost 2 SD from the group’s mean; performance and questionnaire data for this individual were inconspicuous. After excluding this individual from the analysis, all main effects and the interaction of IL-6 remained significant but not the interaction for IL-1β (Table S4 in Supplement 1).

Exclusion of participants using prescribed medication or with CMV-positive status did not change any of the aforementioned results, nor did the exclusion of participants who used drugs by injection. Following the removal of either comorbid alcohol or opiate-dependent individuals from the sample, the valence-by-category-by-group interaction for SCRs was no longer significant.

### Relationships Between Measures of Disgust-induced Change

Only in CDIs we found significant relationships between the different measures of disgust-induced change (i.e., difference scores): In the nonfood condition, the disgust-induced increase in SCRs and response accuracy were significantly correlated (*r* = .48, *p* < .05); in the food condition, CDIs were not aroused during disgust-provocation, and the disgust-induced decrease in SCRs was marginally correlated with the increase in IL-6, as measured at the end of the disgust block (*r* = −.38, *p* = .085). No relationships were identified with self-report measures. In further exploring cocaine users’ responses to food stimuli, we observed significant relationships between disgust-induced decrease in SCRs and overall IL-6 levels (*r* = −.43, *p* < .05) and between disgust-induced change in response latency and the frequency of cocaine use (Spearmen’s *r* = .61, *p* < .001).

## Discussion

The results of the present study provide compelling evidence of a hypersensitivity to stimuli conveying a risk of infection in cocaine-dependent individuals. Concurrent with the notion that feelings of disgust evoke an instant sensory rejection (30), all participants exhibited prolonged response times when presented with disgust-evoking photographs; responses to disgusting nonfood images were particularly slow in the cocaine group (Figure 2A). While viewing the disgusting nonfood pictures, the cocaine group exhibited increased SCRs (Figure 2B), reflecting increased sympathetic tone (61). They also showed a significant increase in the secretion of salivary IL-6 during the disgust provocation (Figure 2C), possibly indicating an anticipatory mechanism to an imminent infectious challenge (25,35,62).

### Potential Mechanisms Underlying a Hypersensitivity to Disgust

At first glance, the upregulated IL-6 response to disgust-evoking stimuli in the cocaine group might be difficult to reconcile with the immunosuppressive effects of cocaine (20,21); however, at closer inspection, this contradiction can be explained by conditioned autonomic responses. Anticipatory immune responses often result from Pavlovian conditioning, that is, they are acquired through repeated pairings of a stimulus with an immunomodulatory agent (25). Consequently, anticipatory responses are observed in individuals with regular exposure to inflammatory challenges, for example, in cancer patients before undergoing their next cycle of chemotherapy (63–65). Analogous conditioned learning may thus underlie the upregulation of salivary IL-6 in CDIs when presented with photographs of infectious sources, irrespective of IL-6 levels in peripheral blood (not measured in this study). Recurrent infections are likely to strengthen associations between infectious stimuli and innate immune responses (25,34). We therefore believe that the abnormal behavioral, autonomic and immune reactions measured in the cocaine group reflect conditioned responses to non-ingestive sources of infection. These conditioned responses may not necessarily be indicative of cocaine users’ compromised immunity.

The autonomic nervous system regulates the process of salivation (66), so IL-6 is increased in response to stress or during septic shock (67), suggesting that the observed IL-6 increase is mediated by nonspecific stress responses. IL-6 is one of the major cytokines that interacts with the sympathetic nervous system and hypothalamic-pituitary-adrenal axis (68). Disgust-provoking photographs have been shown to activate sympathetic tone in healthy individuals, as measured by SCRs (69,70). In the present study, cocaine-dependent individuals exhibited a significant increase in SCRs in response to disgusting, relative to neutral, nonfood photographs (Figure 2B), which was significantly associated with improved detection of disgusting, relative to neutral, nonfood photographs. Since cocaine is known to acutely stimulate sympathetic activity (71,72), one may speculate whether regular cocaine use sensitizes the sympathetic system, rendering individuals more susceptible to psychological or physiological arousal (73,74) and thereby facilitates the secretion of salivary innate immune responses (75).

### Differential Responses to Disgusting Food and Nonfood Stimuli

Hypersensitive disgust-responses in the cocaine group were specific to nonfood stimuli, supporting the notion of different types of disgust. As disgust is elicited by a variety of cues in different contexts, different functions of disgust for survival have been hypothesized (28,30,31). Ingestive disgust evoked by spoiled food may protect against ingesting dangerous toxins, which stands in contrast to feelings of disgust induced by body products, prompting the individual to avoid contact with infectious sources, thereby helping to prevent disease (31). Reduced appetite is a common manifestation of acute infectious illness, which is triggered by the release of pro-inflammatory cytokines such as IL-6 (76,77). The overall increases in salivary IL-6 levels in the cocaine group may be indicative of low-grade inflammation and associated sickness behavior, which could explain the lack of arousal in CDIs while viewing disgusting food images. Importantly, this model of sickness behavior does not contradict the development of hypersensitivity to noningestive stimuli through classical conditioning.

### Methodological Issues and Limitations

Potential limitations of the study include the concurrent use of other drugs in the majority of CDIs, which may also affect immune signaling (15). As polydrug use is extremely common in CDIs (78), it is difficult, if not impossible, to parse out the extent to which our findings are confounded by the immunomodulatory effects of drugs other than cocaine. However, given our aim to elucidate mechanisms that may be associated with increased susceptibility for infection in this population, polydrug use in the present sample may actually serve to enhance the generalizability of our results. Additional measures of cytokine levels in circulating blood would have been desirable to complement the salivary measures. We also acknowledge that other factors associated with the lifestyle of CDIs, such as malnutrition or insomnia (79,80), might modulate inflammation (81,82) but have not been addressed in the present study.

In conclusion, our data indicate that CDIs are more sensitive to cues signaling noningestive sources of infection, which were not related to drug-taking but depicted general scenes involving dirt, decay, wounds, and bodily excrement. Increased physiologic arousal during the viewing of the disgust-evoking cues is likely to have mediated the upregulation of salivary IL-6 in the cocaine group (67,75). We have no evidence to assume that CDIs actively approach these kinds of disgusting and infectious situations; in fact, their hypersensitive reactions suggest that they might have been less successful than their healthy peers at avoiding such risks. Indeed, the observed hypersensitivity in behavioral, autonomic, and immune responses in cocaine users was not reflected in their attitudes towards disgust and hygiene behavior, as one would expect of individuals at risk for infection. This reconciles with the notion of compromised interoception associated with cocaine dependence (83–86). Disruptions in interoceptive processes may have profound implications on general health and well-being if interoceptive feedback is not used to guide behavior. Given that CDIs do not associate their drug use with heightened risk for infection (87), and, as our data suggest, do not act on bodily signals, they are likely to continue exposing themselves to pathogens; as exemplified by continuing to share drug paraphernalia, even if sterile equipment is freely available (88).

Although we did not directly test this hypothesis, our findings suggest that harm-reduction approaches alone are insufficient to reduce the high infection rate associated with cocaine dependence. Education about infection risk and training of interoceptive awareness are potential interventions to be considered. Nonetheless, more research is clearly warranted to break the vicious cycle of infections in addiction.
