# Supplementary material for: Aberrant Disgust Responses and Immune Reactivity in Cocaine-Dependent Men
Source: Biol Psychiatry. 2014 Jan 15;75(2):140–7. doi: 10.1016/j.biopsych.2013.08.004 (PMC3898808; doi:10.1016/j.biopsych.2013.08.004)
Supplement: Supplementary file 1 — Supplementary Material [file mmc1.pdf]

Aberrant Disgust Responses and Immune Reactivity in Cocaine-Dependent Men

Supplemental Information

Supplemental Methods

Study Sample

Cocaine-dependent participants with co-morbid opiate dependence were either prescribed methadone (32%, mean dose: 55 mg ± 17.1 SD) or buprenorphine (9%, mean dose: 3 mg ± 2.6 SD), or were using street heroin on a daily basis (3%). Some cocaine users reported taking prescribed medication, including antidepressants (5%), benzodiazepines (6%), morphine-based pain relief (12%), and amphetamines (2%). Irregular use of antihistamines (2%) and steroids (2%) were reported for the treatment of hay-fever and asthma, respectively.

A minority of healthy participants reported irregular use of antihistamines (2%) and steroids (2%) were reported for treatment of hay-fever and asthma.

Statistical Data Analysis

The Depression Anxiety Stress Scale (DASS-21) total scores and C-reactive protein (CRP)-levels were included as covariates in all group comparisons to obviate potential confounding effects of group differences in mood states and inflammatory markers on immune responses. Both covariates were correlated with the outcome variables, i.e. DASS-21 was correlated with various cytokine levels, in particular the pro-inflammatory cytokines interleukin (IL)-6 (r = -0.41, $p < 0.001$), IL-1beta (r = -0.55, $p = 0.002$) and tumor necrosis factor-alpha (r = -0.38, $p = 0.048$), with trait-disgust on the Disgust Propensity-Sensitivity Scale (DPSS-R) (r = 0.44, $p = 0.019$) and marginally with response latencies on the evocative task (r = -0.37, $p = 0.051$). CRP-levels were significantly correlated with response latency (r = 0.47, $p = 0.010$) and performance accuracy (r = 0.59, $p = 0.006$) on the evocative task; CRP-levels also correlated with IL-6 levels (r = 0.27, $p = 0.032$). The only outcome variable that was not correlated with the DASS-21 or CRP-levels was the Hygiene Inventory (HI-23) total score; subsequent re-analysis with the covariates did not change the results ($t_{59} = -0.47$; $p = 0.642$).

Supplemental Results

Table S1. Ratings of the photographs used in the present study are listed with regard to stimulus category, valence and source. The photographs were selected from a pool of 180 pictures either downloaded from the internet or selected from the International Affective Picture Series (IAPS). To ensure correct valence classification, all pictures were rated for pleasantness, arousal, disgust and nausea on a Likert scale (1 = not, 7 = very) by 15 independent healthy men prior to experimental testing.

Category	Valence	Source	Number	Pleasant	Appetizing	Arousing	Disgusting	Nauseating
Non-food	Neutral	Non-IAPS	19	5.40 (±0.4)	2.01 (±0.39)	2.28 (±0.47)	1.48 (±0.23)	1.43 (±0.27)
Non-food	Neutral	IAPS	11	5.39 (±0.27)	1.83 (±0.28)	2.37 (±0.2)	1.59 (±0.14)	1.50 (±0.19)
Food	Neutral	Non-IAPS	24	5.34 (±0.74)	4.06 (±0.76)	2.80 (±0.46)	1.95 (±0.65)	1.66 (±0.51)
Food	Neutral	IAPS	6	5.16 (±0.68)	3.38 (±0.74)	2.90 (±0.21)	2.18 (±0.76)	1.98 (±0.64)
Non-food	Disgust	Non-IAPS	10	1.73 (±0.32)	1.29 (±0.17)	4.01 (±0.38)	5.47 (±0.40)	4.54 (±0.32)
Non-food	Disgust	IAPS	20	1.81 (±0.43)	1.22 (±0.17)	4.42 (±0.54)	5.67 (±0.56)	4.65 (±0.42)
Food	Disgust	Non-IAPS	30	2.43 (±0.37)	1.43 (±0.19)	3.15 (±0.49)	4.32 (±0.50)	3.76 (±0.48)
Food	Disgust	IAPS	0	¾	¾	¾	¾	¾

Table S2. Demographic and baseline data of the study sample, including the scores of the National Adult Reading Test (NART), Depression Anxiety Stress Scale (DASS-21), Disgust Propensity and Sensitivity Scale (DPSS), and Hygiene Inventory (HI-23). Participants were also asked three closed questions of which the percentage of yes responses are shown below (*Do you get more than three colds a year? Do you find it hard to fight an infection (cold or otherwise)? Do you often take antibiotics more than twice a year?*).

	**Control Group**	**Cocaine Group**	**Comparison**		
	**Mean (±SD)**	**Mean (±SD)**	***t* or *F***	***df***	***p***
Age (years)	37.3 (±10.4)	36.2 (±9.2)	0.46	59	0.650
Verbal Intelligence (NART score)	115.4 (±5.9)	104.1 (±9.9)	27.78	43.8	**<0.001**
Systolic Blood Pressure (mm Hg)	131.7 (±15.7)	132.5 (±15.8)	0.19	59	0.847
Diastolic Blood Pressure (mm Hg)	79.1 (±9.6)	76.7 (±12.4)	0.85	59	0.399
Pulse (rate)	70.3 (±13.5)	66.5 (±11.9)	1.15	59	0.253
DASS-21 Depression (score)	1.5 (±2.0)	7.8 (±5.3)	35.76	1,55	**<0.001**
DASS-21 Anxiety (score)	1.7 (±2.8)	10.9 (±5.3)	33.92	1,55	**<0.001**
DASS-21 Stress (score)	5.0 (±5.0)	15.3 (±9.9)	24.96	1,55	**<0.001**
DPSS Disgust Propensity (score)	14.2 (±3.6)	16.6 (±3.8)	1.17	1,53	0.285
DPSS Disgust Sensitivity (score)	9.7 (±2.7)	11.7 (±3.8)	0.36	1,53	0.552
HI-23 General Hygiene (score)	20.7 (±4.1)	21.9 (±3.9)	0.35	1,53	0.560
HI-23 Household Hygiene (score)	10.9 (±1.4)	10.7 (±1.8)	0.56	1,53	0.875
HI-23 Food-related Hygiene (score)	9.5 (±2.5)	9.7 (±2.3)	0.87	1,53	0.879
HI-23 Hand Hygiene (score)	15.7 (±1.7)	15.7 (±2.0)	0.88	1,53	0.157
HI-23 Personal Hygiene (score)	11.2 (±3.3)	11.1 (±3.0)	0.16	1,53	0.340
More Than Three Colds Per Year (self-report)	6.7%	19.4%	Fisher's exact		0.255
Use of Antibiotics More Than Twice a Year (self-report)	0%	16.1%	Fisher's exact		0.053
Difficulties Fighting an Infection (self-report)	6.7%	35.5%	Fisher's exact		**0.011**

Bolded *p* values indicate significance.

Table S3. Performance data on the evocative task with regard to detection accuracy, response latency and skin conductivity using ANCOVA models.

**Measure**	**Category**	**Valence**	**Group**	**Mean (±SD)**	***F* (category)**	***p* (category)**	***F* (valence)**	***p* (valence)**	***F* (group)**	***p* (group)**	***F* (category x valence x group)**	***p* (category x valence x group)**
Detection accuracy (d')	Non-food	Neutral	Control	0.99 (±0.02)	0.01	0.933	0.04	0.852	0.71	0.403	0.62	0.435
			Cocaine	0.97 (±0.08)								
		Disgust	Control	0.99 (±0.02)								
			Cocaine	0.99 (±0.01)								
	Food	Neutral	Control	0.99 (±0.02)								
			Cocaine	0.99 (±0.02)								
		Disgust	Control	1.00 (±0.01)								
			Cocaine	0.99 (±0.03)								
Response latency (ms)	Non-food	Neutral	Control	1,240.0 (±409.5)	0.01	0.917	7.64	**0.008**	0.03	0.857	4.59	**0.037**
			Cocaine	1,247.5 (±276.3)								
		Disgust	Control	1,452.1 (±387.7)								
			Cocaine	1,591.2 (±408.9)								
	Food	Neutral	Control	1,171.1 (±289.4)								
			Cocaine	1,295.1 (±287.3)								
		Disgust	Control	1,413.2 (±431.7)								
			Cocaine	1,554.0 (±382.5)								
Skin conductance responses (SCRs)	Non-food	Neutral	Control	0.42 (±0.10)	0.14	0.714	0.11	0.746	0.04	0.852	6.27	**0.017**
			Cocaine	0.35 (±0.09)								
		Disgust	Control	0.37 (±0.11)								
			Cocaine	0.39 (±0.15)								
	Food	Neutral	Control	0.38 (±0.10)								
			Cocaine	0.42 (±0.10)								
		Disgust	Control	0.34 (±0.08)								
			Cocaine	0.33 (±0.13)								

Bolded *p* values indicate significance.

Table S4. Changes in salivary cytokine levels at three time points during the evocative task using ANCOVA models.

		Time 1 (arrival)	Time 2 (neutral)	Time 3 (disgust)						
**Cytokines**	**Group**	**Mean (±SD)**	**Mean (±SD)**	**Mean (±SD)**	***F* (time)**	***p* (time)**	***F* (group)**	***p* (group)**	***F* (time x group)**	***p* (time x group)**
INF-g	Control	1.55 (±1.8)	2.51 (±5.1)	3.05 (±6.7)	4.91	**0.014**	1.00	0.321	1.53	0.224
	Cocaine	1.40 (±1.1)	2.50 (±3.2)	3.87 (±6.8)						
IL-10	Control	1.56 (±1.2)	2.25 (±2.2)	2.32 (±2.2)	5.49	**0.012**	0.07	0.788	0.22	0.805
	Cocaine	1.31 (±0.8)	1.71 (±1.2)	2.02 (±1.7)						
IL-12p70	Control	0.52 (±0.1)	0.58 (±0.3)	0.61 (±0.5)	0.35	0.651	0.29	0.595	0.76	0.437
	Cocaine	0.53 (±0.1)	0.63 (±0.4)	0.74 (±0.9)						
IL-1b	Control	3.84 (±3.2)	5.97 (±5.6)	5.60 (±4.6)	6.40	**0.004**	1.87	0.177	2.40	0.102
	Cocaine	3.23 (±3.3)	5.95 (±7.1)	7.49 (±8.0)						
IL-6	Control	6.73 (±6.4)	9.06 (±9.5)	8.21 (±7.4)	5.90	**0.006**	8.77	**0.005**	4.55	**0.017**
	Cocaine	11.68 (±23.1)	16.78 (±24.4)	21.85 (±36.2)						
IL-8	Control	10.79 (±12.4)	13.70 (±13.6)	13.30 (±14.2)	1.69	0.190	0.00	0.995	0.85	0.429
	Cocaine	8.66 (±8.0)	14.32 (±11.8)	16.11 (±12.5)						
TNF-a	Control	5.14 (±8.6)	6.70 (±12.8)	7.34 (±13.0)	4.72	**0.021**	0.88	0.352	0.91	0.378
	Cocaine	2.05 (±2.6)	3.32 (±4.0)	4.25 (±4.6)						

IL, interleukin; INF, interferon; TNF, tumor necrosis factor.
Bolded *p* values indicate significance.
